# Tubular Duplication of the Midgut Presenting with Acute Abdomen and Hematochezia: A Case Report from Northern Tanzania

**DOI:** 10.1155/2018/2858723

**Published:** 2018-01-24

**Authors:** Ayesiga M. Herman, David Msuya, Mwemezi Kaino, Hilary Shilanaiman, Joachim Magoma, Christian Issangya, Jay Lodhia, Murad Tarmohamed, Jeremia Pyuza, Patric Amsi, Alex Mremi, Ronald Mbwasi, Nuru Letara

**Affiliations:** ^1^Department of General Surgery, Kilimanjaro Christian Medical Center, P.O. Box 3010, Moshi, Tanzania; ^2^Kilimanjaro Christian Medical University College, P.O. Box 2240, Moshi, Tanzania; ^3^Department of Anesthesia, Kilimanjaro Christian Medical Center, P.O. Box 3010, Moshi, Tanzania; ^4^Department of Pathology, Kilimanjaro Christian Medical Center, P.O. Box 3010, Moshi, Tanzania; ^5^Department of Pediatrics, Kilimanjaro Christian Medical Center, P.O. Box 3010, Moshi, Tanzania

## Abstract

Enteric duplication is one of the rare malformations affecting the small intestine more than the other parts of the gastrointestinal tract. It poses a challenge in diagnosis due to nonspecific symptoms that may mimic other pathologies. Furthermore, the management options including total resection, mucosal striping, and internal drainage of the duplicate depend on the presentation of the patient, site, and length of the involved bowel. We present the first documented case of enteric duplication in Tanzania, a 3-year-old male, who was found to have a 90 cm long jejunoileal duplicate. We discuss the presentation and management offered.

## 1. Introduction

Enteric duplication is one of the rare malformations usually presenting with variety of symptoms including abdominal pain, intestinal obstruction, perforations, and gastrointestinal bleeding [1]. The duplication may be cystic or tubular with the midgut being more affected followed by the foregut and the hindgut being the least [1–3]. There are theories explaining the possible cause of duplication, but all remains speculative [4–6]. We report a case of a 3-year-old boy who presented to us with the history of abdominal pain and distension, with symptoms of gastrointestinal bleeding and anemia, and then later found to have duplicate small intestines at laparotomy.

## 2. Case Presentation

A 3-year-old male patient presented with a history of abdominal pain and vomiting for two days and a history of rectal bleeding for 4 months. The abdominal pain was generalized associated with vomiting. The mother reported on and off episodes of passing black tarred stools since when he was four months of age. Those episodes occurred at least once a year, lasting for few days, with spontaneous remission.

There was no history of constipation, diarrhea, or hematemesis.

On examination, the child was sick looking, malnourished, and conscious with conjunctiva and palmar pallor. He had a temperature of 36.9°C, and no finger clubbing. His heart rate was 140 beats per minute with a respiratory rate of 24 breaths per minute and oxygen saturation of 98% in room air.

Per abdomen, it was distended and tender with muscle guarding on palpation. Bowel sounds were present with reduced frequency and pitch. On digital rectal examination, the rectum was loaded with fecal matter, and no mass palpable and no bleeding were noted on removal of the gloved finger. Other systems had normal findings.

**Figure 1:**
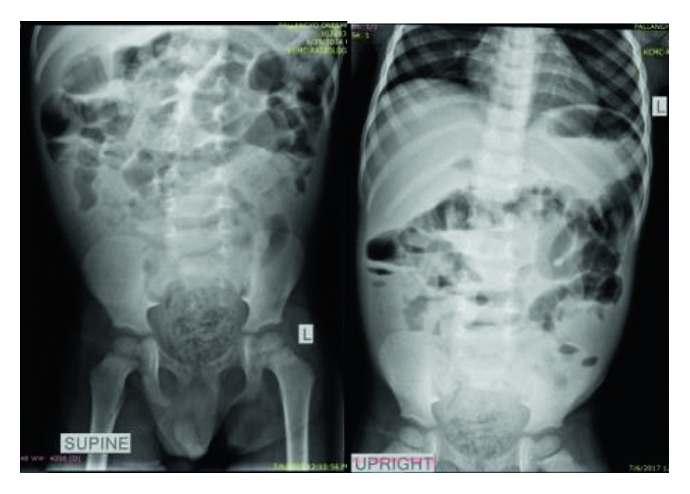
Plain abdominal X-rays (erect and supine) showing proximal small bowel loop dilatation, gaseous with some air fluid levels, while the distal part being dense with the rectum loaded with fecal matters.

The plain abdominal X-ray done on admission showed proximal dilatation of small bowel loops, gaseous with some air fluid levels, while the distal part being dense with the rectum loaded with fecal matters (Figure 1).

The abdominal ultrasound showed gaseous abdomen with dilated bowel loops and minimal pelvic collection.

The patient's blood group was O positive, with hemoglobin of 6.6 g/dl microcytic hypochromic cells. Also, the full blood count showed leukocytosis of 17.65 × 10^9^/l predominantly neutrophils (71.5%).

He had low sodium levels of 129 mmol/l (135–145 mmol/L) and slightly high potassium levels of 5.14 mmol/l (3.5–5.0 mmol/L) with normal chloride levels. Serum protein levels were low (34.2 g/L) with hypoalbuminemia (16.23 g/L). The Widal test was negative.

The patient was diagnosed to have peritonitis, with anemia due to lower versus upper gastrointestinal bleeding and malnutrition.

**Figure 2:**
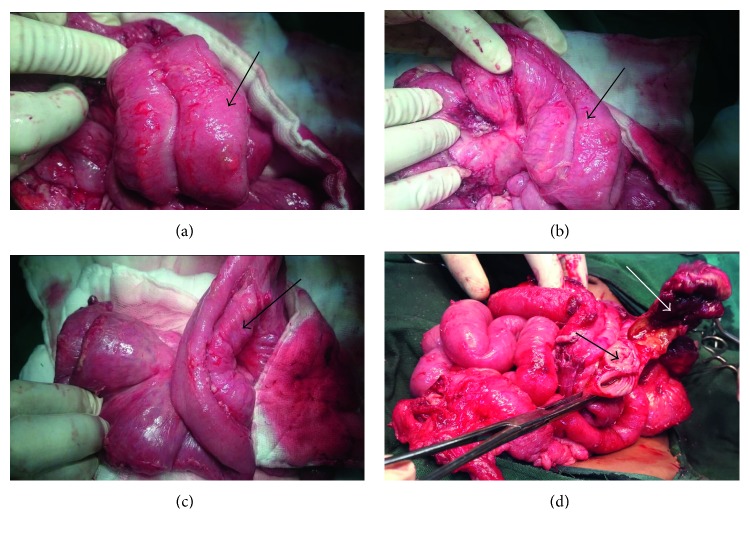
Tubular duplicate of the small intestine: (a), (b), and (c) show duplicate intestine (black arrows) with the two bowel loops sharing the mesentery, and (d) shows the double lumen (black arrow) with a perforation at the site of confluence (white arrow).

The decision for exploratory laparotomy was made after optimizing the patient. A supraumbilical transverse incision was used to access the abdominal cavity. Intraoperative findings were multiple immature adhesions with pus pockets. The omentum was adhered to the pelvic region. There was a tubular duplication of the small intestine starting at 15 cm from the ligament of Treitz with the proximal part of the duplicate ending blindly and the distal forming a confluence with the terminal ileum (about 20 centimeters from ileocecal junction). The duplicate bowels shared the mesentery spanning the length of about 90 centimeters with a perforation at the mesenteric border of the confluence (Figure 2(d)). The appendix was in retrocaecal position and perforated at its base.

**Figure 3:**
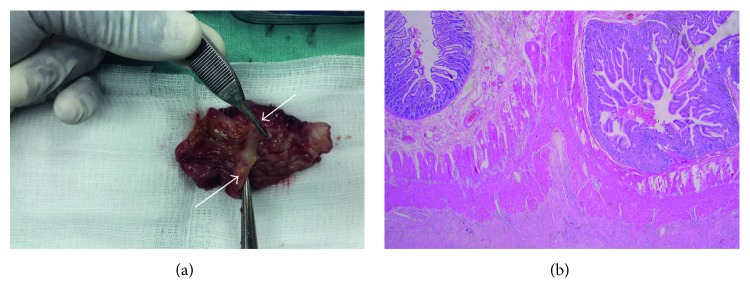
(a) Resected portion of the duplicate, showing the confluence of the two lumens (black arrows) and the dividing septum (white arrow). (b) Histology slide of (a), showing the double lumen and a septum with muscularis propria.

Resection of the perforated segment involving the confluence of the two lumens was done followed by end-to-end anastomosis to the terminal ileum. A side-to-side anastomosis for proximal duplicate lumens was done to promote drainage and prevent blind loop syndrome. Appendicectomy was also done. The histology of the resected part of the confluence showed double lumen with the septum containing the muscularis propria (Figure 3(b)).

The patient was nursed in intensive care unit postoperatively. He received blood transfusion, proton-pump inhibitors, antibiotics, and nutritional supplements. On the 7th day after surgery, he developed surgical site infection with wound dehiscence and leakage from the proximal side to side anastomosis with the duplicate lumen. Repair was done with application of retention sutures.

The patient improved and fared well after the second surgery and was discharged 3 weeks later. He was followed up after discharge for six months in the clinic and had no any new complaints.

## 3. Discussion

Enteric duplication is a condition of rare occurrence. The incidence is about 1 out of 10,000 live births with most of the duplication affecting the small intestine (jejunum and ileum) [7, 8]. Most of the patients usually adults are found incidentally while being investigated for other pathologies, whereas young individuals may present with myriad symptoms [3, 7]. Several theories concerning the etiology are put forth; however, none have been able to describe the syndrome in totality [4, 5].

The presentation of the patients may vary depending on the type of the duplication, whether circular or tubular. Also, the location of the duplicate plays a big role in the symptomatology. The common listed presenting symptoms are vomiting, abdominal distension, and abdominal pain [9]. Other symptoms include lower gastrointestinal bleeding and abdominal swelling due to mass effect following intestinal obstruction or intussusception [9]. The diagnosis of enteric duplication poses a challenge due to nonspecific symptoms, which may mimic other conditions. However, diagnostic tools such as abdominal ultrasound, barium studies, and CT scan may help establish a clue especially in low-resource settings [10]. Other modalities like the use of laparoscopy may aid in diagnosis and management in some selected cases with duplicate intestine [11], although their availability may be limited in most resource-limited settings.

Despite the wide range of differential diagnoses which are possible in pediatric patients, a high index of suspicion of duplicate intestine in this population is important. This will allow targeted diagnostic tests and timely intervention. Our patient's initial problem was never identified and hence led to progression of the disease process. His initial symptoms constituted gastrointestinal bleeding, with severe anemia, and later developed abdominal pain with signs of peritonitis. It is with this regard some authors have advocated to always have high index of suspicion of duplicate intestine when making differential diagnosis in pediatric patients with hematochezia [2].

The management options for duplicate intestine include surgical resection of the duplicate or internal drainage [3]. If the involved segment is short, it is removed with the adjacent normal bowel [8]. Other scholars have suggested mucosal striping through sequential transverse incisions in the duplicate bowel or drainage into the stomach for anomalies involving longer segment of the bowel [8, 12]. Some authors have suggested that, in case of presence of heterotopic mucosa, the drainage into the adjacent lumen is not advised due to the risk of perforation [8]. From these arguments, it seems that the preferred approach is to resect all the affected bowel segments with reconstruction of the remaining part for bowel continuity. However, the challenge in this approach as faced by other authors is the fact that resection of a long segment of the bowel will result in short bowel with nutritional deficiency leading to retarded growth [12]. In this situation, doing an enteroenterostomy or marsupialization with the duplicate being left in place to avoid resecting too much of normal bowel is advised [13–15]. Our patient had approximately 90 cm loop of small bowel involved with the duplicate. In this instance, internal drainage of the duplicate segment was preferred.

The surgical procedure in children with enteric duplication aims at elimination of symptoms as compared to the one that may be done in adults, which in addition aims at total removal of the duplicate intestine due to the documented risk of developing neoplasia [6, 10, 13–16]. In a review by Orr and Edwards, most of the neoplastic changes with duplicate intestine involved adult individuals with age ranging from 38 years to 64 years [10]. These neoplastic changes, however, in addition to age seem to affect more of the large intestine [6, 10, 13]. Some reviewed reports have suggested the large bowel duplication to be termed as premalignant condition, though this may need more appraisals of this rare condition [10]. However, other parts of the alimentary tract involving the stomach duplication cyst and small intestine have also been reported with regard to neoplastic changes [10, 16].

## 4. Conclusion

It is important to think of intestinal duplication as part of differential diagnosis when dealing with pediatric patients presenting with hematochezia. Furthermore, the management of the patient should consider the site and length of the duplicate segment of the bowel for good outcomes.
